# Retrospective Analysis of a Modified Irrigation Method for Nasopharyngeal Carcinoma Patients With Post-Radiation Nasopharyngeal Necrosis

**DOI:** 10.3389/fonc.2021.663132

**Published:** 2021-04-29

**Authors:** Yun Xiao, Shiyi Peng, Yiqiang Tang, Honghui Xie, Min Huang, Jing Wang, Xiaochang Gong, Jingao Li

**Affiliations:** ^1^ Department of Radiation Oncology, Jiangxi Cancer Hospital of Nanchang University, Nanchang, China; ^2^ NHC Key Laboratory of Personalized Diagnosis and Treatment of Nasopharyngeal Carcinoma, Jiangxi Cancer Hospital of Nanchang University, Nanchang, China; ^3^ Faculty of Medicine, Nanchang University, Nanchang, China

**Keywords:** post-radiation nasopharyngeal necrosis, nasopharyngeal carcinoma, retrospective analysis, nasopharyngeal irrigation, magnetic resonance imaging

## Abstract

**Purpose:**

Post-radiation nasopharyngeal necrosis (PRNN) is one of the most serious late effects of nasopharyngeal carcinoma (NPC) after radiotherapy. Standard conservative treatments are not always effective, and this study sought to investigate the feasibility of modified nasopharyngeal irrigation in the treatment of PRNN.

**Methods:**

Between September 2011 and September 2018, 113 NPC patients with pathologically or radiologically diagnosed PRNN were analyzed retrospectively. All patients received the traditional conservative treatments of debridement of the necrotic tissues guided by an endoscope and systematic antibiotic therapy partly guided by culture results. The patients were divided into two groups according to the irrigation method used: traditional and modified groups. Modified irrigation used an irrigation device made by our hospital, guided by endoscopy, while the patients in the traditional irrigation group used a nasopharyngeal irrigation pot to wash the nasopharynx by themselves each day.

**Results:**

Survival was affected by ICA (internal carotid artery) exposure, necrosis grade, and re-irradiation, but only ICA exposure and re-irradiation were found to be independent prognostic factors. The modified irrigation had a significantly more positive effect on the recovery rates of patients with mild- and moderate-grade PRNN than did traditional irrigation. The 2-year overall survival (OS) of the 113 patients was 68.4%. The modified irrigation was associated with better OS in the mild- and moderate-grade groups, in the one-course radiotherapy group, and in the low-risk group (according to the 2017 system).

**Conclusions:**

More intense modified irrigation under the physicians control may be an effective treatment for PRNN, especially mild- and moderate-grade, one-course radiotherapy, or low-risk PRNN.

## Introduction

Nasopharyngeal carcinoma (NPC) is a common malignant head and neck cancer in Southeast Asia and southern China, and the mainstay of treatment for NPC is radiotherapy (1, 2). With developments in radiotherapy techniques, intensity-modulated radiotherapy (IMRT) has become widely used in clinical practice, and the 5-year overall survival (OS) rate of patients with NPC has significantly improved to 85% (3). However, in the radiation field, complications are inevitable, especially in patients undergoing re-irradiation. Post-radiation nasopharyngeal necrosis (PRNN) is one of the most serious later effects of nasopharyngeal carcinoma after radiotherapy (4) Post-radiation necrosis is induced by multiple factors (5) and might be an indicator of poor prognosis for patients with NPC (6). The incidence of nasopharyngeal necrosis after initial traditional radiotherapy in patients with nasopharyngeal carcinoma is 0.2% to 0.3%, but with IMRT, it is 1.5% (7), and the rate of nasopharyngeal necrosis and hemorrhage in second-course radiotherapy is as high as 40.6% (8). PRNN is often accompanied by headache, foul nasal odor, and epistaxis, which serious damage to the quality of patients lives (9). PRNN can even lead to fatal consequences, including massive hemorrhage, infection, and cachexia (9, 10).

Radiation and trauma are important risk factors for PRNN. Nasopharyngeal mucosal barrier function is weakened after mucosal injury, increasing the risk of infection. Many conservative treatment strategies, such as hyperbaric oxygen, daily nasopharyngeal irrigation, endoscopic debridement of the necrotic tissues, intravenous nutrition, and systematic antibiotic therapy, have been tested clinically, but the outcomes are disappointing (9, 10). In recent years, it has been reported that radical endoscopic necrotomy followed by reconstruction with nasal flap (ENNF) may have a better prognosis than conservative treatment for patients with PRNN (11). However, such surgical techniques are highly demanding and difficult to promote (12). Traditional nasopharyngeal irrigation that a large amount of fluid rapidly rinses the necrotic surface is of great significance in the treatment of nasopharyngeal carcinoma necrosis and may control infection better and increase the intense sensation of the treatment (13, 14).

Since 2015, patients with partial nasopharyngeal necrosis are received modified nasopharyngeal irrigation, which was performed under nasopharyngeal mirror. In this study, we analyzed clinical and magnetic resonance imaging (MRI) features, treatment methods, and outcomes in 113 NPC patients with PRNN to identify a feasible and effective treatment method to improve the survival time of patients with PRNN.

## Materials and Methods

### Patients

The studies involving human participants were reviewed and approved by the Ethics Committee of Jiangxi Cancer Hospital of Nanchang University. The patients/participants provided their written informed consent to participate in this study.

This study analyzed the records of 113 NPC patients (77 males and 36 females) with pathologically or radiologically diagnosed PRNN from September 2011 to September 2018. Patients chose the irrigation method according to their own wishes. According to the different irrigation treatment methods, the patients were divided into traditional and modified irrigation groups. There were 62 cases in the traditional irrigation group and 51 in the modified irrigation group. There were 26 cases of internal carotid artery (ICA) exposure, 41 cases of severe necrosis, and 21 cases of second-course radiotherapy (**Table 1**). All patients received plasma EBV-DNA detection, and the results were negative. All patients had varying degrees of headache, foul nasal odor, and epistaxis. The latent period between the last irradiation and the onset of symptoms ranged from 0 to 79 months, with a median of 4 months. The modified irrigation was performed since May 2014. On the final follow-up date (30th July 2019), the median follow-up was 30.5 months and the median age was 57 years (range, 3175 years).

**Table 1:** Clinical characteristics of 113 patients with post-radiation nasopharyngeal necrosis (PRNN).

Clinical characteristics	Modified irrigation group	Traditional irrigation group	*P*-value
	n=51 (%)	n= 62 (%)	
Gender			0.84
Female	34(66.7)	43(69.4)	
Male	17(33.3)	19(30.6)	
Age (years)			0.542
60	37(72.5)	41(66.1)	
>60	14(27.5)	21(33.9)	
T stage			0.016
T1+T2	0(0)	7(11.3)	
T3+T4	51(100)	55(88.7)	
NPC stage			0.22
I+II	1(2.0)	5(8.1)	
III+IV	50(88.0)	57(91.9)	
Cycles of chemotherapy			0.036
No	8(15.7)	22(35.5)	
2	22(42.3)	16(25.8)	
>2	21(42.0)	24(38.7)	
Re-irradiation			0.39
Yes	11(21.6)	10(16.1)	
No	40(78.4)	52(83.9)	
ICA exposure			0.043
Yes	7(13.7)	19(30.6)	
No	44(86.3)	43(69.4)	
Grade of PRNN			
Mild	10(19.6)	9(14.5)	0.096
Moderate	28(54.9)	25(40.3)	
Severe	13(25.5)	28(45.2)	
Risk classification			0.317
Low	37(72.5)	38(61.3)	
Intermediate	10(16.1)	20(32.3)	
High	4(7.84)	4(6.4)	

NPC, nasopharyngeal carcinoma; ICA, internal carotid artery.

The exclusion criteria were (1) pathology-confirmed cancerous ulcers and (2) patients who underwent surgery or intervention before irrigation.

### Radiotherapy

Twenty-one patients with recurrent tumors received re-irradiation, and all patients received IMRT or volumetric modulated arc therapy (VMAT). All patients were immobilized in the supine position with a thermoplastic mask. After administration of intravenous contrast material, 3mm CT slices were acquired from the head to a level 2cm below the sternoclavicular joint. For one course of radiotherapy, 66 to 70 Gy was applied to the PTV of the Gross tumor volume of nasopharynx (GTVnx), 60 Gy to the PTV of clinical target volume (CTV) 1 (i.e., high-risk regions), 54 to 56 Gy to the PTV of CTV2 (i.e., low-risk regions), and 64 to 70 Gy to the PTV of the GTVnd for the metastatic cervical lymph nodes in 30 to 35 fractions. For a second course of radiotherapy, 60 to 70 Gy was applied to the PTV of the GTVnx and 50 to 54 Gy to the PTV of CTV1 in 27 to 35 fractions.

### Magnetic Resonance Imaging

All patients underwent evaluation using MRI with a 1.5-T system (Signa, General Electric). The area from the suprasellar cistern to the inferior margin of the sternal end of the clavicle was scanned with a head-and-neck combined coil.

### Diagnosis of PRNN

**Figure 1:**
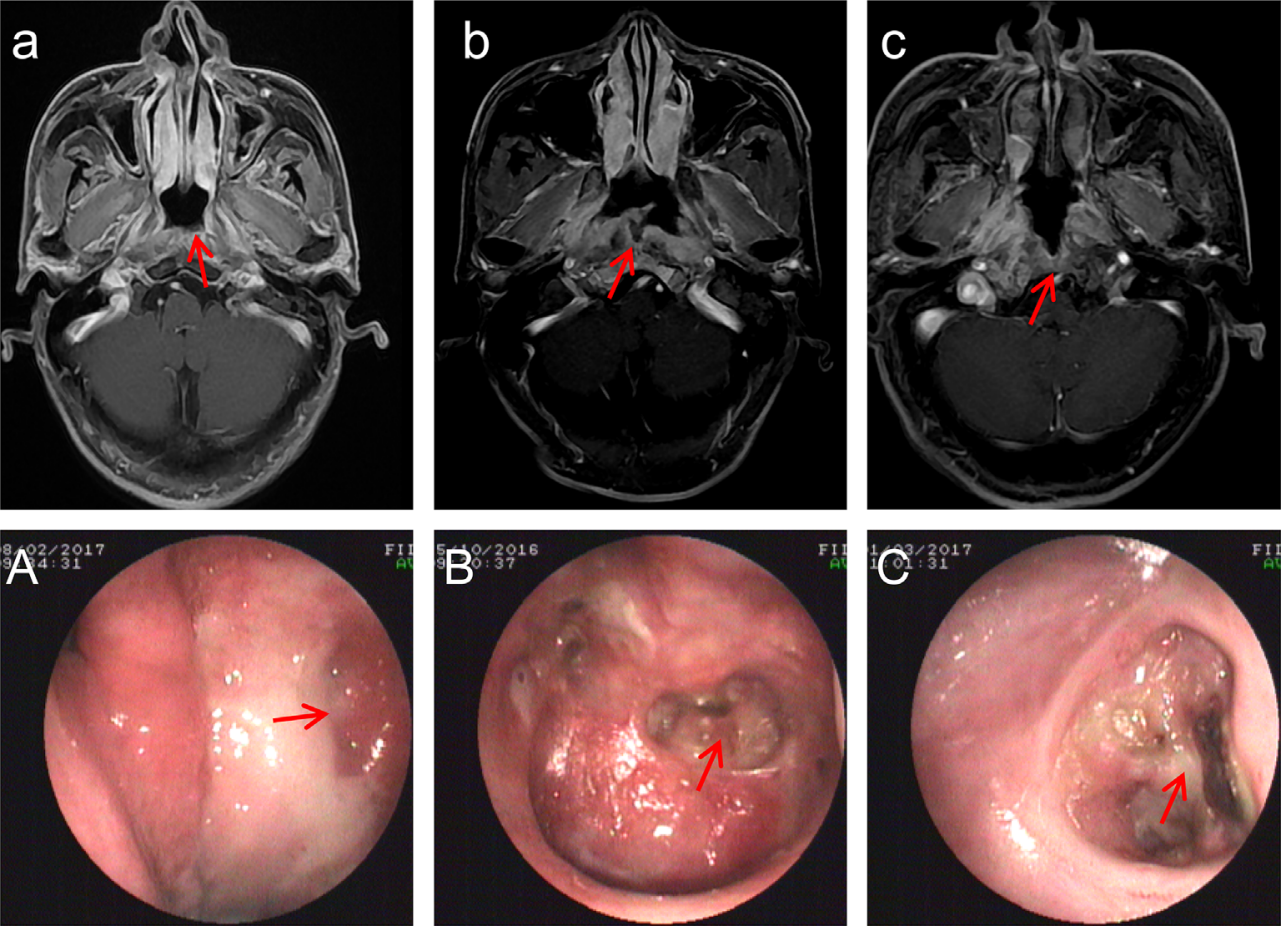
MRI and endoscopic images with post-radiation nasopharyngeal necrosis (PRNN). **(a)** Transverse, contrast-enhanced T1-weighted magnetic resonance imaging (MRI) of mild-grade PRNN shows discontinuous nasopharyngeal mucosa lines. **(A)** Endoscopic image with mild-grade PRNN. **(b)** Transverse, contrast-enhanced T1-weighted MRI of moderate-grade PRNN shows discontinuous mucosa lines and non-enhanced areas. **(B)** Endoscopic image with moderate-grade PRNN shows a superficial ulcer. **(c)** Transverse, contrast-enhanced T1-weighted MRI of severe-grade PRNN shows invasion of the skull base bone. **(C)** Endoscopic images with moderate-grade PRNN show a deep ulcer. The red arrow points to the lesion.

Nasopharyngoscopy and MRI imaging are used to diagnose PRNN. All patients had previously undergone radiotherapy. The PRNN was graded into three degrees according to the characteristics of the endoscope and MRI examinations (**Figure 1**). A mild-grade PRNN was necrosis confined to the mucous and interrupted mucosal line that could be seen with MRI. A moderate-grade PRNN was necrosis invading the surrounding soft tissue, but not exceeding the muscle tissue; there were also non-enhanced areas on contrast-enhanced T1-weighted MRI images. A severe-grade PRNN was an ulcer exceeding the muscle tissue to the skull base bone; these were reported in a previous study (9). If the tumor recurrence was suspected during flushing and review, the biopsy was taken in time for exclusion. Biopsies were taken from 37 patients suspected of recurrent NPC, and the results confirmed PRNN.

### Treatment of PRNN

All patients received debridement treatment of the necrotic tissues guided by endoscope and systematic antibiotic therapy partly under the guidance of nasopharyngeal secretion drug sensitivity test.

Patients in the traditional irrigation group used a common nasopharyngeal irrigation pot to wash the nasopharynx three times a day with warm boiled water or light saltwater (500ml). The specific operation was that the patient squeezed the nasopharyngeal irrigating pot by hand, so that the irrigating fluid flowed into the nasopharynx slowly, and then flowed out from the oral cavity. The opposite side was rinsed in the same way. The patients needed to persist for lifetime.

**Figure 2:**
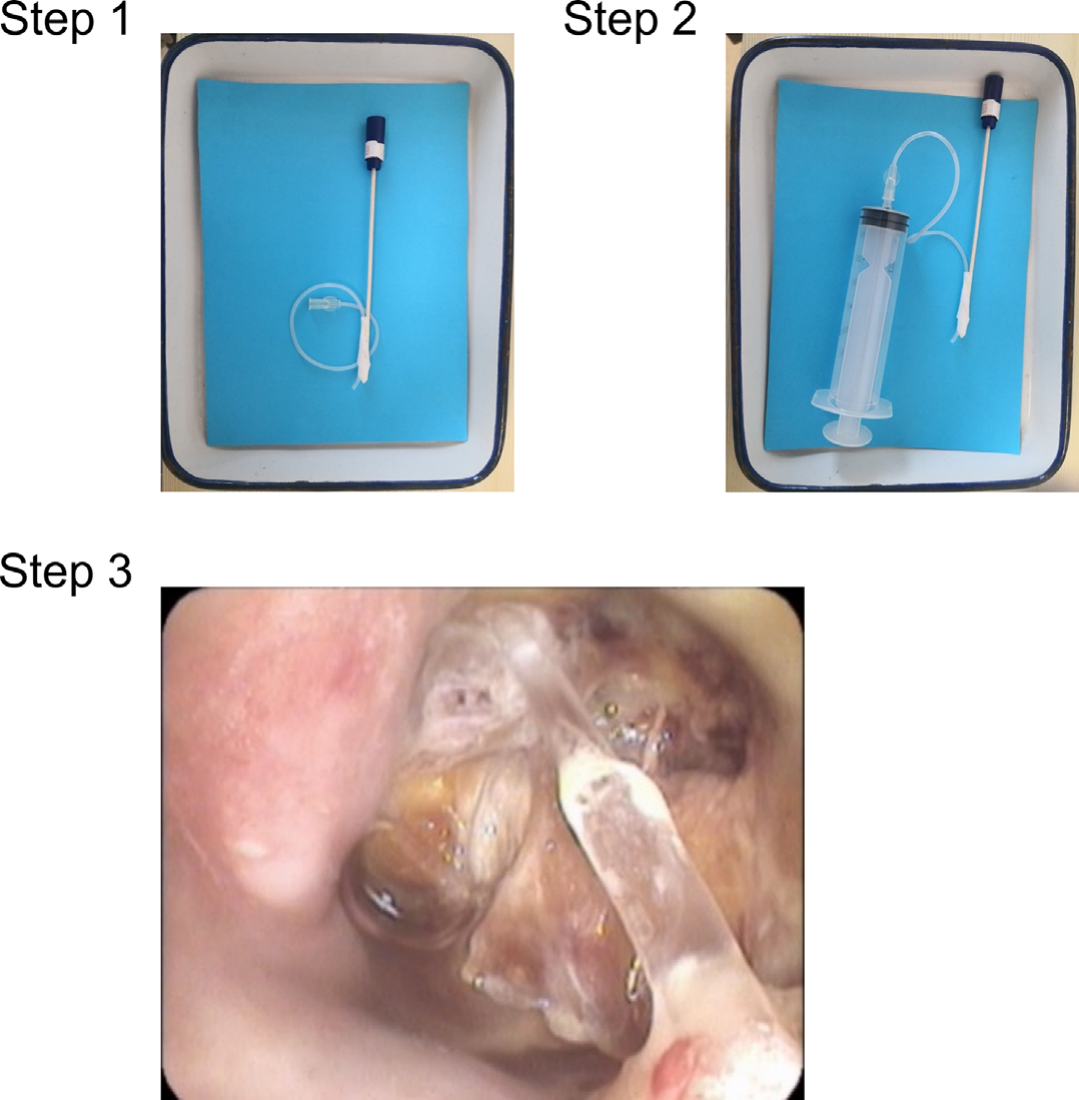
Modified irrigation device and usage. Step 1: a connecting tube of the infusion is attached to a wooden throat swab stick. Step 2: a 50-ml syringe is connected to the connecting tube to form the self-made nasopharyngeal irrigation device. Step 3: the device is used to irrigate the PRNN lesion.

In addition to rinsing by themselves every day like the patients in the traditional group, patients in the modified irrigation group also received direct irrigation with an electronic nasopharyngoscopy operated by a physician. The specific modified irrigation method is to let the patient take a sitting position, enter the nasopharyngoscopy from one side of the nasal cavity, insert the homemade nasopharyngeal irrigation device (**Figure 2**) to the nasopharyngeal ulcer on the other side of the nasal cavity, and use a 50-ml syringe to rinse under the direct vision of the nasopharyngoscopy. If the patient received one time modified irrigation, the patient would correspondingly reduced one time traditional irrigation on the same day. At the beginning of the treatment (3 months), due to lack of experience, 8 patients in the modified irrigation group were irrigated every other day from Monday to Friday. They were found to have good tolerance. Then all patients in modified irrigation group were irrigated 5 times a week on working days. The commonly used 1000ml rinsing solutions included metronidazole, saline, 1.5% aquae hydrogen peroxide, and KangFuXin solution. When the mucous membrane at the ulcer was repaired and healed and the clinical symptoms such as headache, odor, bleeding, etc. were relieved, the patients could stop the modified irrigation and be discharged from the hospital. After discharge, the patients continued to rinse by themselves for lifetime like the patients in the traditional irrigation group. The median numbers of the modified irrigation times in the modified irrigation group were 13 times (range, 5175) for patients in the whole group, 14 times (range, 6175) for patients with second-course radiotherapy, and 13 times (range, 497) for patients with the first-course radiotherapy.

### Statistical Analysis

Our primary outcome was OS, which was calculated from the date when PRNN was diagnosed to the date of death or of the last follow-up. Statistical analysis was performed using the SPSS 23.0 software package. Survival curves for OS were analyzed by the KaplanMeier method and compared using log-rank tests. Univariate and multivariate analyses were performed using the Cox proportional hazards model. Univariate Cox regression analysis was performed to identify potential prognostic factors for PRNN. Multivariate Cox regression analysis was performed to distinguish independent factors for PRNN from the variables with statistical significance in the univariate analysis. Differences in categorical data were assessed by chi-squared tests. All statistical tests were two-sided, and a *p*-value of less than 0.05 was considered statistically significant.

## Results

### Clinical and MRI Characteristics of Patients With PRNN

There were significant differences in T stage, ICA exposure, and the number of chemotherapy cycles between the modified irrigation group and the traditional irrigation group. There were no significant differences in gender, age, NPC stage, re-irradiation, grade of PRNN, risk classification between the modified irrigation group and the traditional irrigation group (**Table 1**).

Contrast-enhanced T1-weighted MRI images showed defects in the nasopharyngeal mucosa and may have shown the non-enhanced soft tissues. Defects in the parapharyngeal space and involvement of the ICA or skull base bone could also be observed.

**Figure 3:**
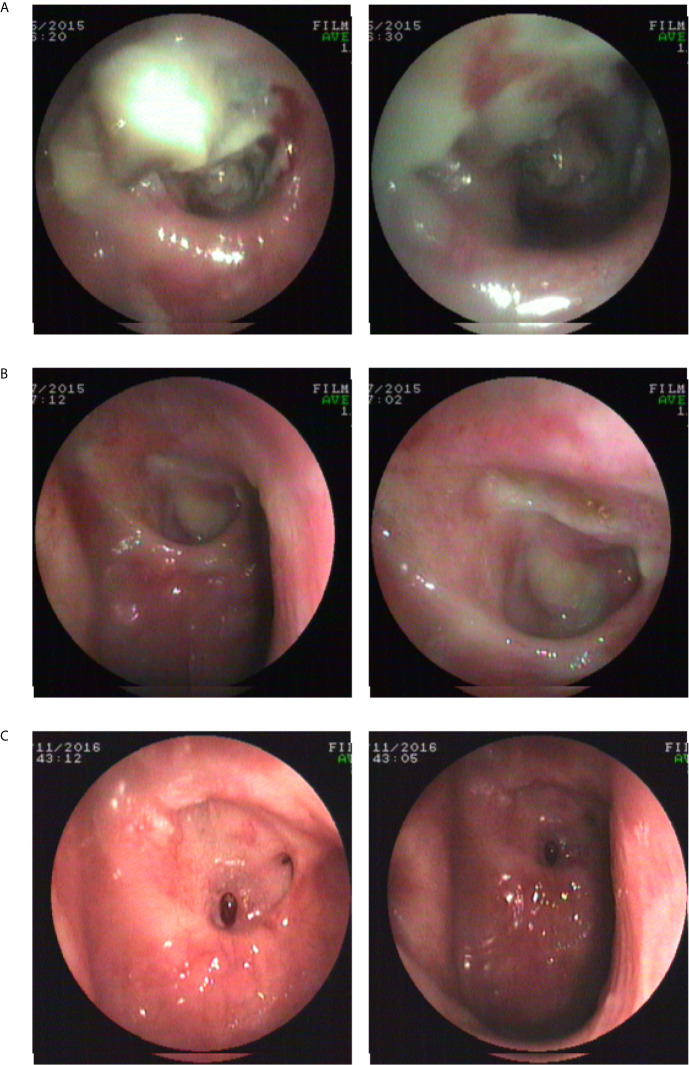
Endoscopic images before and after modified irrigation. **(A)** Before modified irrigation, the necrosis is located in the nasopharyngeal cavity. **(B)** Three months after modified irrigation treatment, the necrotic secretions and tissues appear to have decreased. **(C)** One year after modified irrigation treatment, a small ulcer remains in the posterior wall of the nasopharynx, but necrotic secretions and tissues disappear.

Three months after modified irrigation, the necrotic secretions and tissues appeared to have decreased. One year after modified irrigation, small ulcers remained in the posterior wall of the nasopharynx, but the necrotic secretions and tissues had disappeared (**Figure 3**).

### Risk Factors for the Survival of Patients With PRNN

The results of the univariate survival analyses shown in **Table 2** suggested that the ICA exposure, necrotic grade, skull base osteoradionecrosis, and re-irradiation were associated with poor prognosis. The results of multivariate Cox modeling also shown in **Table 2** indicated that the ICA exposure and re-irradiation were independent prognostic factors.

**Table 2:** Univariate and multivariate analyses of variables associated with overall survival (OS).

Characteristics	Univariate analysis		Multivariate analysis	
	Hazard ratio (95% CI)	*P*-value	Hazard ratio (95% CI)	*P*-value
Gender	0.863(0.458-1.627)	0.649		
Age	1.685(0.909-3.126)	0.098		
T stage	0.910(0.558-1.487)	0.326		
NPC stage	1.977(0.271-14.44)	0.502		
Cycles of chemotherapy	0.831(0.574-1.203)	0.326		
Grade of PRNN	2.071 (1.2733.369)	0.003	2.715 (0.8368.814)	0.096
Osteoradionecrosis	2.203 (1.1984.053)	0.017	4.300 (0.1101.675)	0.224
ICA exposure	3.305 (1.7836.127)	0.000	2.062 (1.0124.200)	0.046
Re-irradiation	3.206 (1.6606.195)	0.001	2.079 (1.0154.256)	0.045
Irrigation method	0.518 (0.2591.037)	0.063		

NPC, nasopharyngeal carcinoma; PRNN, post-radiation nasopharyngeal necrosis; ICA, internal carotid artery.

### Patient Recovery Rates of Patients With PRNN

Of the 113 cases, 62 recovered and 43 died, 23 due to massive nasopharyngeal bleeding (including 18 of the 26 patients [69.2%] with ICA exposure), 19 from exhaustion, and one from a cerebral hemorrhage. The recovery rate from mild-grade PRNN was 63.2% (12/19), from moderate-grade was 50.9% (27/53), and from severe-grade was 17% (7/41). Patients who received modified irrigation had a higher recovery rate than those in the traditional irrigation group (52.9% [27/51] vs. 30.6% [19/62], *p*=0.016). Further subgroup analysis showed that the modified irrigation had a significantly more positive effect on the recovery rates of patients with mild- and moderate-grade PRNN than traditional irrigation (65.8% vs. 41.1%, *p*=0.017).

### Overall Survival of Patients With PRNN

**Figure 4:**
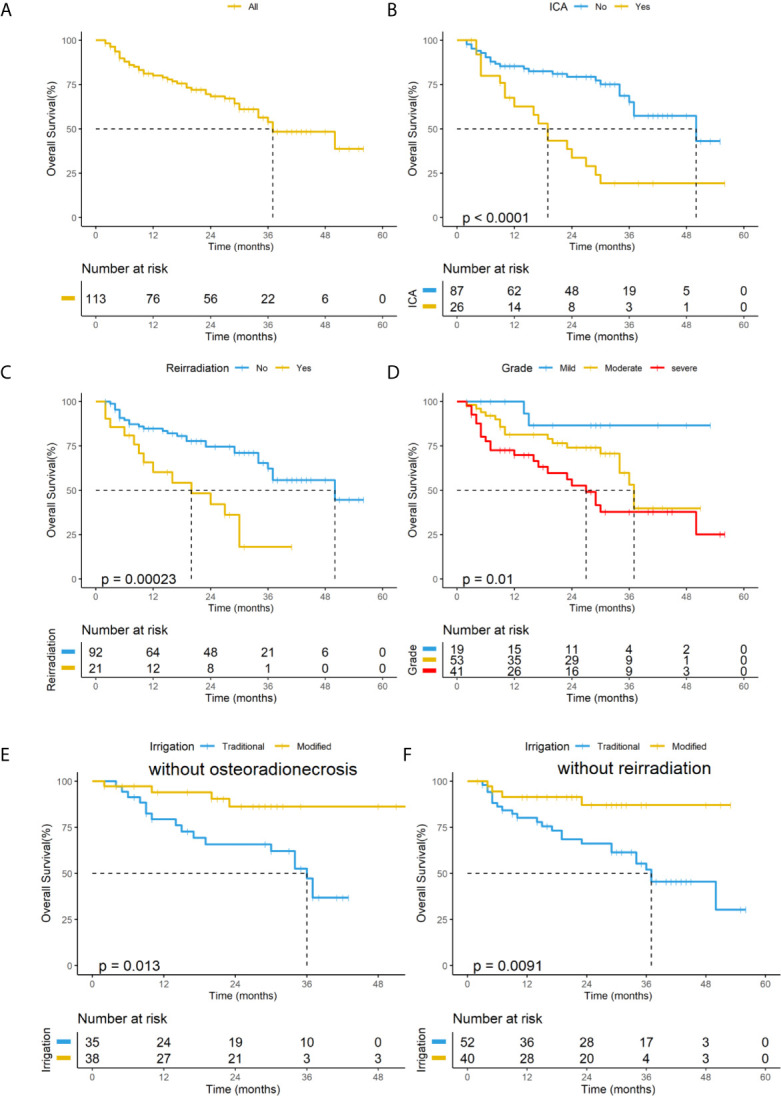
KaplanMeier curves of overall survival for all patients with post-radiation nasopharyngeal necrosis (PRNN). **(A)** all patients. **(B)** patients stratified by ICA exposure. **(C)** patients stratified by re-irradiation. **(D)** patients stratified by the grade of PRNN based on the standard classification system. **(E)** subgroup analysis for patients with mild- and moderate-grade PRNN based on to the standard classification system. **(F)** subgroup analysis for patients without re-irradiation. ICA, internal carotid artery.

The 2-year OS rate of the 113 patients with PRNN was 68.4% (**Figure 4A**). The 2-year OS rate of the ICA exposure group was 33.8%, and that of the no exposure group was 79.3% (**Figure 4B**). The 2-year OS rate of the one-course radiotherapy group was 84.8%, and that of the second-course radiotherapy group was 42.2% (**Figure 4C**). The 2-year OS rate of the mild-grade necrosis group was 86.7%, of the moderate-grade group was 74%, and the severe-grade group 52.7% based on the standard classification system (**Figure 4D**). For patients in the whole group, the improved irrigation could not improve the OS compared with the traditional irrigation. However, further subgroup analysis showed that the modified irrigation was associated with better OS in the mild- and moderate-grade group (without osteoradionecrosis group) and in the without re-irradiation group than traditional irrigation (86.4% vs. 54.6%, *p*=0.013; 87% vs. 66.2%, *p*=0.0091) (**Figures 4E, F** **)**.

**Figure 5:**
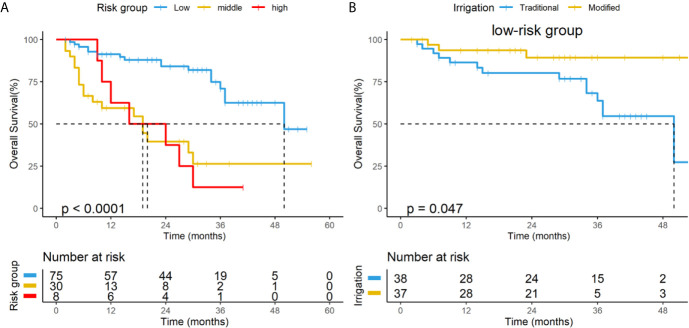
**(A)** KaplanMeier curves of overall survival for all patients in the subgroups based on the new risk classification system. **(B)** Subgroup analysis for patients in the low-risk group based on the new risk classification system.

Multivariate analysis in this study found that ICA and re-radiotherapy are independent prognostic factors for OS, which are consistent with the risk factors in the new staging systems proposed by Yang etal. in 2017 (11). So we also investigated the prognosis of patients based on new staging systems. **Figure 5A** shows the KaplanMeier curves for the OS of patients within each stage according to the 2017 system; the OS curves of the low-, intermediate-, and high-risk groups were statistically significant (P<0.001). Further subgroup analysis showed that modified irrigation was associated with better OS in the low-risk group than traditional irrigation (*p*=0.047) (**Figure 5B**).

## Discussion

This study examined the impact of two irrigation treatments on survival in patients with PRNN. The main findings are as follows: ICA exposure and re-irradiation were independent prognostic factors for PRNN; the modified irrigation had a significantly more positive effect on the recovery rates of patients with mild- and moderate-grade PRNN than did traditional irrigation; the modified irrigation increased 2-year OS of patients and was associated with better OS in the mild- and moderate-grade groups, in the one-course radiotherapy group, and in the low-risk group (according to the 2017 system).

Concerning differential diagnosis, patients with a recurrent or residual NPC may show similar signs and symptoms, especially when tumor necrosis occurs, and nasopharyngeal endoscope, MRI, and pathological examination of the nasopharynx are helpful for the diagnosis of nasopharyngeal necrosis. In patients with nasopharyngeal necrosis, a large number of filament necrotic secretions or tissues in the nasopharynx accompanied by soft tissue defects were observed under nasopharyngeal endoscopy, and some ulcerations were deep, showing ICA or bone exposure (15). Contrast-enhanced T1-weighted MRI images showed discontinuous nasopharyngeal mucosa lines and non-enhanced areas mixed with tiny air bubbles (16), but recurrent or residual tumors always displayed enhanced tissues. The contrast-enhanced T1-weighted MRI images of the PRNN patients enrolled in this study presented defects in the nasopharyngeal mucosa and parapharyngeal space and involvement of ICA or the skull base bone.

Although the exact mechanism is unknown, irradiation, trauma, and infection are considerable risk factors for PRNN. The damaged mucosal barrier also increases the risk of infection, which leads to prolonged necrosis and greatly affects the prognosis and quality of life of patients with NPC. The final cause of death in patients with PRNN is usually massive bleeding and systemic failure (9). The same is true of the cases in this study, and patients were observed to die because of massive nasopharyngeal bleeding, exhaustion, and cerebral hemorrhage.

Prognosis in patients with PRNN is generally poor, especially in those with ICA exposure, skull base osteoradionecrosis, and re-irradiation. Yi etal. retrospectively analyzed 28 patients with PRNN, and nine of the 13 who had ICA exposure (69.2%) died of massive hemorrhage. Furthermore, multivariable logistic regression analysis indicated that ICA exposure was the only independent prognostic factor (9). Similarly, Chen etal. retrospectively analyzed the clinical and imaging features of 67 NPC patients with PRNN; of the 33 patients with ICA exposure, 24 (72.7%) died and suggested that MRI findings are diagnosis-specific helping the doctor to stage the severity of the necrosis (10). Yang etal. retrospectively analyzed 276 patients with PRNN and found that 49.4% of the 81 patients with ICA exposure died of sudden massive hemorrhage and that the mortality risk forpatients who received re-irradiation increased by 42.8%. Our data showed that 69.2% of patients with ICA exposure died from massive bleeding; given the high mortality rate of patients with ICA exposure, ICA embolization is essential after a negative internal carotid artery occlusion test.

Re-irradiation and ICA exposure decreased patients 2-year OS significantly. Through the multivariate analysis, we found that re-irradiation and ICA exposure were independent prognostic factors for PRNN. A new risk classification system was proposed: low risk, without ICA exposure or re-irradiation history; intermediate risk, with ICA exposure or re-irradiation history; and high risk, with both ICA exposure and re-irradiation history. Based on this risk classification system, the 2-year OS of the low-risk group was 64.8%, the intermediate-risk group was 45.1%, and the high-risk group was 22.5%, which are statistically significantly different (*p*<0.001).

ENNF improved survival in the low-risk group (*p*=0.001) but exhibited only a trend toward significance in the intermediate-risk (*p*=0.081) and high-risk (and *p*=0.066) groups. ENNF may lead to better survival outcomes than conservative management in PRNN patients, but more studies are needed to validate this new risk classification system (11). Our results also suggest that risk classification systems should be reconsidered.

Based on the standard classification system (9), PRNN was divided into three degrees, mild, moderate, and severe, and there were significant differences in survival in the three groups. There were also significant differences in survival in the three groups (low, intermediate, and high risk) of the new risk classification system (11), but the differences were smaller than in the standard classification system. Therefore, we believe that the new risk classification system of PRNN is feasible and appropriate for prognosis.

In subgroup analysis, modified irrigation improved OS in patients in the mild-grade, moderate-grade, and low-risk (without ICA exposure or second-course radiotherapy) groups, and thus appears to be a valuable method for treating PRNN. The modified irrigation had limited efficacy in the re-irradiation group, which may be related to the nasopharyngeal necrosis after re-irradiation being the most severe grade, with many patients unable to irrigate consistently and timely for financial and other personal reasons. PRNN patients need to be diagnosed and treated in a timely manner, as the cure rate is low and the risk of death high once necrosis develops to the severe grade.

When using the traditional irrigation method, the amount of irrigation fluid to reach the nasopharyngeal cavity is small and the impulse is small, which cannot effectively remove the necrotic secretions from the nasopharyngeal ulcer, and it is difficult to achieve the expected effect. When using our modified irrigation device, the flushing head is placed on the lesion site, which would then be flushed with plenty of fluid. The modified irrigation can better control local infection by effectively removing necrotic secretions, creating good conditions for the self-repair of patients ulcers, and alleviating patients symptoms more quickly.

There are some limitations to this study. First, not all patients had a biopsy pathology confirmation that the PRNN was non-cancerous, although it was clinically and radiologically considered to be so; second, this study is a single-center retrospective study, and selective bias cannot be avoided; third, this study did not compare the quality of life of patients between the two treatment methods; in addition, this study is a non-randomized controlled study and the effect of patients self-irrigation cannot be guaranteed. Therefore, this study cannot determine whether the benefit of modified irrigation is due to the increase in irrigation intensity or the irrigation technology itself. The next step is a prospective randomized controlled study to determine the benefit of the modified irrigation for the treatment of PRNN.

## Conclusion

More intense modified irrigation under the physicians control may be favorable for PRNN patients with mild-grade and moderate-grade PRNN, those who have undergone only one course of radiotherapy, or those in the low-risk group. And it can be performed in most hospitals easily. These results suggest a feasible and effective method to treat PRNN.

## Data Availability Statement

The raw data supporting the conclusions of this article will be made available by the authors, without undue reservation.

## Ethics Statement

The studies involving human participants were reviewed and approved by the Ethics Committee of Jiangxi Cancer Hospital of Nanchang University. The patients/participants provided their written informed consent to participate in this study.

## Author Contributions

YX collected the data, followed up patients, and drafted the manuscript. SP, YT, HX, MH, and JW collected the data. XG designed the study and collected the data. JL designed the study. All authors read and approved the final manuscript.

## Conflict of Interest

The authors declare that the research was conducted in the absence of any commercial or financial relationships that could be construed as a potential conflict of interest.
